# Supplementary material for: Challenges Facing Undergraduate Medical Education in Ambulatory Care Clinics at Tertiary Care Hospitals
Source: Healthcare (Basel). 2022 Mar 8;10(3):496. doi: 10.3390/healthcare10030496 (PMC8951531; doi:10.3390/healthcare10030496)
Supplement: Supplementary file 1 [file healthcare-10-00496-s001.zip › Figure S1. Student Survey.pdf]

Figure S1. Students Survey.

Challenges Facing Undergraduate Medical Education in Ambulatory Care Clinics at Tertiary Care Hospitals

Greetings everyone,

The journey of going through medical school is full of obstacles. One way to help passing these obstacles is by discovering them first! From this point of view, we got our research question.

We are conducting a study to investigate the challenges our students in years 4 and 5 face in the outpatient department (OPD) at KFSHRC and KKUH, as a tertiary care hospital.

We strongly encourage you to fill this survey, and would highly appreciate your input! Filling the survey will not take you 2-3 mins.

PI: Dr. Fahad Alsohaibani
Email: [redacted]

*Required

1. Gender: *

Mark only one oval.

- ( ) Female
- ( ) Male

2. Year: *

Mark only one oval.

- ( ) 4th
- ( ) 5th

[redacted]

3. University: *

Mark only one oval.

- Alfaisal University
- King Saud University

4. As a medical student, with whom do you prefer attending clinics ? *

Mark only one oval.

- Consultant
- Fellow
- Senior Resident
- Junior Resident

5. During your training in 4th/5th year, what are the barriers and challenges for medical students have proper and efficient outpatient education ? *

Mark only one oval.

- Institution related
- Faculty Related
- Patient Related
- Student Related
- Other: ____________________

Please rank the following factors that interfere with outpatient education in order of importance

6. Environmental/Institution related: *

Mark only one oval per row.

	Inadequate distribution of student in ambulatory clinics	Lack of structured teaching objectives	Inappropriate or small clinics rooms	Restrictions due to COVID-19
Very important	⬭	⬭	⬭	⬭
Fairly important	⬭	⬭	⬭	⬭
Important	⬭	⬭	⬭	⬭
Slightly important	⬭	⬭	⬭	⬭

7. Faculty Related: *

Mark only one oval per row.

	Inadequate supervision and teaching by faculty	Lack of time for teaching by doctor due to intense patient agenda	Inappropriate or absence of feedback	Fear of losin private patients
Very important	⬭	⬭	⬭	⬭
Fairly important	⬭	⬭	⬭	⬭
Important	⬭	⬭	⬭	⬭
Slightly important	⬭	⬭	⬭	⬭

8. Patient Related: *

Mark only one oval per row.

	Patients refusal to be seen by medical students	Lack of suitable patients for teaching	No follow-up/continuity of care for cases attended
Very important	⬭	⬭	⬭
Important	⬭	⬭	⬭
Slightly important	⬭	⬭	⬭

9. Student Related: *

Mark only one oval per row.

	Increasing number of students attending ambulatory clinics	Lack of student's commitment and interest in learning	No additional information gained compared to inpatient setting	No enough time to atten the clinics
Very important	⬭	⬭	⬭	⬭
Fairly important	⬭	⬭	⬭	⬭
Important	⬭	⬭	⬭	⬭
Slightly important	⬭	⬭	⬭	⬭

10. What do you think are the best clinics you have attended during your medical school ? *

Tick all that apply.

- [ ] ENT
- [ ] Family Medicine
- [ ] Internal Medicine and IM Subspecialties
- [ ] OBGYN
- [ ] Ophthalmology
- [ ] Orthopaedics
- [ ] Paediatrics
- [ ] General Surgery
- [ ] Neuroscience
- [ ] Psychiatry

Other: [ ] ______________________

11. Do you support involving medical students in virtual clinics ? *

Mark only one oval.

	1	2	3	4	5	
Strongly Agree	◯	◯	◯	◯	◯	Strongly disagree

[redacted]

[redacted]
