# Supplementary material for: Challenges Facing Undergraduate Medical Education in Ambulatory Care Clinics at Tertiary Care Hospitals
Source: Healthcare (Basel). 2022 Mar 8;10(3):496. doi: 10.3390/healthcare10030496 (PMC8951531; doi:10.3390/healthcare10030496)
Supplement: Supplementary file 1 [file healthcare-10-00496-s001.zip › Figure S2. Faculty Survey.pdf]

[redacted]

Figure S2. Faculty Survey.

Challenges facing Undergraduate Medical Education in Ambulatory Care Clinics at Tertiary Care Hospitals

Faculty Version

The journey of going through medical school is full of obstacles. One way to help passing these obstacles is by discovering them first! From this point of view, we got our research question.

We are conducting a study to investigate the challenges our students in years 4 and 5 face in the outpatien department (OPD) at KFSHRC and KKUH, as a tertiary care hospital.

We strongly encourage you to fill this survey, and would highly appreciate your input! Filling the survey will not take you 2-3 mins.

PI: Dr. Fahad Alsohaibani
Email: [redacted]

---

*Required

1. Gender: *

Mark only one oval.

- ◯ Female
- ◯ Male

[redacted]

2. Age (years): *

Mark only one oval.

- ( ) Below 30
- ( ) 30-39
- ( ) 40-49
- ( ) 50-59
- ( ) 60 and above

3. University: *

Mark only one oval.

- ( ) Alfaisal University
- ( ) King Saud University

4. Academic Title: *

Mark only one oval.

- ( ) Professor
- ( ) Associate Professor
- ( ) Assistant professor
- ( ) Lecturer
- ( ) Clerkship director

5. Speciality : *

Mark only one oval.

- Internal Medicine (and IM subspecialties)
- Surgery (and surgical subspecialties)
- Paediatrics
- OBGYN
- Neurosciences
- Ophthalmology
- Family medicine
- ENT
- Orthopaedics
- Psychiatry
- Other: ____________________

6. Do you feel comfortable if a medical students attend Ambulatory Care Clinics (ACC) with you

Mark only one oval.

	1	2	3	4	5	
Strongly agree	⬭	⬭	⬭	⬭	⬭	Strongly disagree

7. Do you think outpatient experience is important for medical students and it should be integrated in the curriculum? *

Mark only one oval.

- ⬭ Yes
- ⬭ No

Faculty Perspective: Ambulatory Care Clinics (ACC) Learning Experience

8. From your experience, what are the main challenges/barrier to have proper and efficient medical student education in outpatient settings? *

Mark only one oval.

- ⬭ Environment/institutional-related
- ⬭ Faculty-related
- ⬭ Patient-related
- ⬭ Student-related
- ⬭ Other: ______________________

Please rank the following factors that interfere with outpatient education in order of importanc from 1 to 4, where 1 is the most important and 4 is the least important.

9. A. Environment/institutional-related: *

Mark only one oval per row.

	Lack of institutional support	Inappropriate or small clinics rooms	Inadequate structuring and distribution of student in OPD	Inadequate financial incentives for academic staff	Restriction due to COVID-19
First	◯	◯	◯	◯	◯
Second	◯	◯	◯	◯	◯
Third	◯	◯	◯	◯	◯
Fourth	◯	◯	◯	◯	◯
Fifth	◯	◯	◯	◯	◯

10. B. Faculty-related: *

Mark only one oval per row.

	Insufficient time for teaching due to intense patient agenda	Lack of training/retraining for faculty to teach medical students in OPD	Not integrated as part of my current KPI	Not feeling comfortable to hav students in my clinic
First	◯	◯	◯	◯
Second	◯	◯	◯	◯
Third	◯	◯	◯	◯
Fourth	◯	◯	◯	◯

[redacted]

11. C. Patient-related: *

Mark only one oval per row.

	Patients refusal to be seen by medical students	Lack of suitable patients for teaching	No follow-up/continuity of care for cases attended	Fear of patien dissatisfactio
First				
Second				
Third				
Fourth				

12. D. Student-related: *

Mark only one oval per row.

	Lack of student’s commitment and interest in learning	Increasing numbers of students
First		
Second		

13. Do you support involving medical student in virtual clinics?

Mark only one oval.

	1	2	3	4	5	
Strongly agree						Strongly disagree

[redacted]

[redacted]
